# Dual-emissive solid-state histidine-stabilized gold nanoclusters for applications in white-light generation†

**DOI:** 10.1039/d3na00555k

**Published:** 2023-09-14

**Authors:** Markus Zetes, Alexandru-Milentie Hada, Milica Todea, Luiza Ioana Gaina, Simion Astilean, Ana-Maria Craciun

**Affiliations:** a Nanobiophotonics and Laser Microspectroscopy Center, Interdisciplinary Research Institute in Bio-Nano-Sciences, Babes-Bolyai University 42 T. Laurian Str. 400271 Cluj-Napoca Romania ana.gabudean@ubbcluj.ro; b Faculty of Physics, Babes-Bolyai University 1 M. Kogalniceanu Str. 400084 Cluj-Napoca Romania; c Nanostructured Materials and Bio-Nano-Interfaces Center, Interdisciplinary Research Institute in Bio-Nano-Sciences, Babes-Bolyai University 42 T. Laurian Str. 400271 Cluj-Napoca Romania; d Department of Molecular Sciences, Faculty of Medicine, Iuliu Haţieganu University of Medicine and Pharmacy Cluj-Napoca 400349 Romania; e Research Center on Fundamental and Applied Heterochemistry, Faculty of Chemistry and Chemical Engineering, Babes-Bolyai University 11 A. Janos Str. 400028 Cluj-Napoca Romania

## Abstract

The majority of present-day white-light emitting devices (WLEDs) are built upon the use of rare-earth elements, which have a short supply, are expensive and can become extremely toxic. Thus, in this work, we synthesized an eco-friendly, efficient and cheap white-light emitting material (WLEM) based on solid-state histidine-stabilized gold nanoclusters (His-AuNCs), obtained through the lyophilization of microwave-synthesized photoluminescent His-AuNCs. Their morphological and structural characterization was followed by thorough evaluation of their intrinsic solid-state photoluminescence properties *via* steady-state and time-resolved fluorescence spectroscopy and microscopy, at multiple excitation wavelengths. A white-light emission was observed under UV light excitation due to the two-band broad emission, with maxima at 475 and 520 nm, covering a large area of the visible spectrum. In order to evaluate the purity of the white-light emission we calculated the chromaticity coordinates, at different wavelengths, and displayed them on a CIE (Commision Internationale d’Eclairage) diagram. An excellent value of (0.36, 0.33) was found at 420 nm excitation, which falls within the range of pure white-light emission. Moreover, the His-AuNCs show great photo- and thermo-stability, thus proving their ability to perform as a reliable WLEM with potential use in the development of eco-friendly WLEDs.

## Abstract 2

Photo- and thermo-stable dual-emissive solid-state histidine-stabilized gold nanoclusters demonstrate appealing warm white-light emission capabilities suitable for WLED applications.

## Graphical abstract

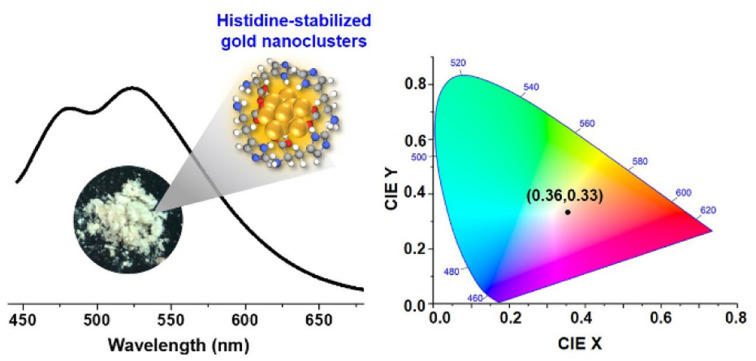

## 1. Introduction

White-light emitting devices (WLEDs) have achieved great brightness levels, efficiency, stability and other great characteristics for commercialization over time. They are heavily used in the production of displays, automotive headlamps, room-lamps, *etc.* A common approach toward white-light generation was reported by Wang *et al.*,^1^ who characterized WLEDs as phosphor atoms that are excited by blue or near-UV irradiation, typically known as down-conversion of light. Unfortunately, there are two main concerns with this approach: firstly, down-converting filters and commercial phosphors contain rare-earth elements, such as europium, terbium, and yttrium.^2^ Rare-earth elements are extremely exploited in different industries leading to a very low supply world-wide, while their recycling procedures could be very toxic for humans and the environment.^3^ Secondly, long exposure to the blue and UV components of a WLED system can cause extreme harm to vision and circadian rhythms of humans.^4,5^ Considering these drawbacks of conventional WLEDs, the development of novel, reliable, eco-friendly and energy efficient white-light emitting materials (WLEMs) is a great challenge among researchers. There have been numerous reports on nanoclusters (NCs) as viable and reliable WLEMs. For example, Tanwar *et al.*.^6^ reported white-light emission from a mixture of silicon quantum dots and gold nanoclusters (AuNCs), while Barman *et al.*^7^ reported white-light generation in a composite of carbon dots and dye-encapsulated bovine serum albumin protein-capped AuNCs. In this context, AuNC-based WLEMs can be promising candidates for reducing the problems caused by conventional WLEDs. Nowadays, AuNCs have gained increased interest among researchers because they are a new class of extremely small nanomaterials with amazing optical properties, such as intrinsic tunable photoluminescence (PL), large Stokes shift, non-toxicity and great photostability.^8^ They are extremely small in dimension (1–3 nm) and can be green-synthesized using template-assisted approaches in the presence of proteins,^9^ peptides,^10^ amino acids,^11^*etc.* A very important feature of AuNCs is that their emission is tunable and the spectral position of the emission band can be manipulated by varying the capping ligand and the amount of gold salt.^12,13^ This property opens them up to a great number of applications in areas like: sensing,^14^ imaging,^15^ catalysis,^16^ optoelectronics,^17^*etc.* Another remarkable feature of AuNCs is that they preserve their PL properties in different states of matter such as colloidal solution or solid-state powder.^15,18^ Huang *et al.* demonstrated that solid-state AuNCs play a huge role in white-light emission due to their stability and emission properties.^19^ Therefore, the necessity of a novel, more efficient and affordable WLEM together with the excellent excitation-dependent and photostable PL properties of AuNCs might be the perfect match to achieve this goal.

Herein, we prove the appealing white-light emissive properties of solid state photoluminescent histidine-stabilized AuNCs (His-AuNCs), as efficient, eco-friendly and stable WLEM, a reliable alternative to the currently employed materials based on rare-earth phosphors or other elements. The obtained His-AuNCs, with an average size of 2.9 ± 0.3 nm, were optically and structurally characterized. Their elemental and structural composition was investigated *via* X-ray photoelectron spectroscopy (XPS), nuclear magnetic resonance (NMR), Fourier transform infrared spectroscopy (FT-IR) and mass spectrometry while steady-state fluorescence spectroscopy and fluorescence lifetime imaging microscopy (FLIM) assays performed at different excitation wavelengths revealed the behavior of their photoluminescence. In particular, solid-state His-AuNCs exhibit a dual-emission with bands localized at 475 and 520 nm, excitation-dependent behavior and fluorescence lifetimes in the 2–3 ns range. Their broad dual-band photoluminescence emission covers a large area of the visible spectral region enriching them with white-light emission properties, as also proved by the image captured from the lyophilized powder under UV light. The purity and relevance of the emitted white-light was evaluated using the Commision Internationale d’Eclairage (CIE) diagram.^6^ For example, under 420 nm excitation, the chromaticity coordinates were found to be (0.36, 0.33), close to those of an ideal white-light source. Additionally, His-AuNCs preserve their WLE properties at high temperatures (up to 150 °C) and after 1 h of continuous irradiation, demonstrating their high resistance to temperature and high photostability. The fabricated novel, green, efficient and extremely photo- and thermo-stable WLEM could have a great impact on a large field of industrial applications and potential to slowly outperforming the traditional WLEDs which are health and environmental hazards.

## 2. Experimental details

### 2.1 Materials

l-Histidine (His) and hydrogen tetrachloroaurate(iii) trihydrate (HAuCl_4_·3H_2_O) of analytical grade were purchased from Sigma-Aldrich. Ultrapure water with a resistivity of 18 MΩ cm used for the preparation of the solutions was obtained with a Milli-Q Millipore purification system (Merck, Massachusetts, USA).

### 2.2 Synthesis and lyophilization of His-AuNCs

All the glassware used in this work was carefully cleaned with aqua regia (HCl/HNO_3_, volume ratio 3 : 1) and rinsed thoroughly with ultrapure water before use. The His-AuNCs were synthesized using a novel method recently published.^20^ Briefly, His (0.1 M, 3 ml) was mixed with HAuCl_4_ (10 mM, 1.5 ml) in a G10 sealable microwave vessel and the mixture was introduced into a microwave reactor, where the probe was irradiated in pulses of 850 W power for 30 minutes at 90 °C and 12 000 rpm. The use of microwaves significantly shortens the synthesis procedure to only 30 minutes making the synthesis more attractive and less time-consuming than room-temperature approaches which take considerably more time. Additionally, as previously shown,^20^ His-AuNCs obtained using microwaves exhibit a four times stronger PL emission, at the same excitation, compared to His-AuNCs obtained at room temperature. Afterwards, the His-AuNCs were purified by centrifugation (6000 rpm, 10 min) using a 10 kDa Satorious centrifugal concentrator (Hettich, Germany) to eliminate the surplus of His and unreduced ions. The as-obtained His-AuNCs were placed in a freezer for 24 h prior to the lyophilization process. Afterwards, the frozen solution was placed in a Biobase BK-FD Series vacuum freeze dryer at −60 °C, and after 24 h it presented itself in a solid state.

### 2.3 Instrumentation

His-AuNCs were prepared in a microwave reactor (Monowave 300, Anton Paar). HR-TEM images were acquired with a Tecnai TEM microscope (G2 F20 X-TWIN) at 200 kV accelerating voltage. Confocal FLIM images and fluorescence lifetime decay curves of the His-AuNCs in the solid state were obtained using a MicroTime200 confocal time-resolved fluorescence microscope system from PicoQuant (Berlin, Germany), under excitation at 375, 405, 485 and 520 nm using pulsed laser diodes operating at 40 MHz. The signals were spatially and spectrally filtered using a 50 μm pinhole and corresponding long-pass emission filters from Chroma (HQ405LP, HQ430LP, and HQ510LP) and Semrock (FF01-519LP). A VL-215.C UV lamp with two 15 W UV tubes at 254 nm was used to analyze the white-light emission of the His-AuNCs solid powder. All photos were taken using a mid-range smartphone camera. The fluorescence spectra of His-AuNCs in the solid state were measured using a JASCO FP6500 spectrofluorometer (Tokyo, Japan) with 1 nm spectral resolution, equipped with a 150 W Xenon lamp as the excitation source and solid sample attachment (FDA 430). The fluorescence spectra of His-AuNCs were obtained with excitation and emission bandwidths fixed at 3 nm. NMR spectra were recorded at room temperature on Bruker Avance instruments (1H/13C: 600 MHz/150 MHz), in solution (deuterated solvents D_2_O) using tetramethylsilane as the internal reference. XPS spectra were recorded with a SPECS PHOIBOS 150 MCD system (Berlin, Germany) employing a monochromatic Al-Kα source (*hν* = 1486.6 eV), a hemispherical analyzer, a multichannel detector and a charge neutralization device. The samples were fixed on double-sided carbon tape and care was taken to ensure that the sample particles covered the tape. The experiments were performed by operating the X-ray source with a power of 200 W, while the pressure in the analysis chamber was in the range of 10^−9^–10^−10^ mbar. The binding energy scale was charge referenced to C 1s at 284.6 eV. The elemental composition was determined from survey spectra acquired at a pass energy of 60 eV. High resolution spectra were obtained using an analyzer pass energy of 20 eV. Charge neutralization was used for all samples. Analysis of the data was carried out with Casa XPS software. A Shirley background was used for all curve-fitting along with the Gaussian/Lorentzian product form (70% Gaussian and 30% Lorentzian). Thermogravimetric analysis (TGA) was performed using a DTG-60H Shimadzu derivatograph (Kyoto, Japan). The sample was heated constantly from 25 °C up to 650 °C. Fourier transform infrared spectroscopy (FT-IR) spectra of His and His-AuNCs were recorded on a Brucker Alpha II spectrometer between 400 and 4000 cm^−1^ using the attenuated total reflectance (ATR) sampling technique. The mass spectra (EI-MS) were recorded on a Shimadzu QP2010+, under the conditions of direct sample introduction, electron impact, and 70 eV.

## 3. Results and discussion

### 3.1 Optical and morphological characterization

**Fig. 1:**
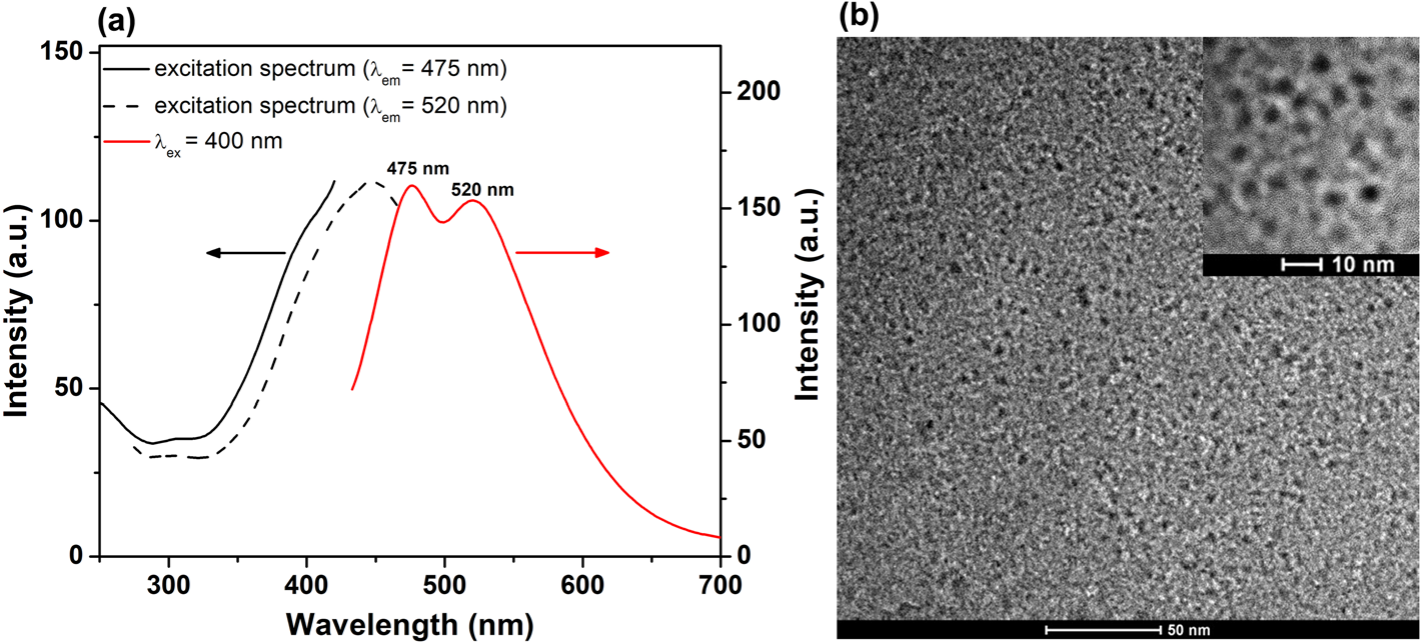
(a) Excitation spectra (*λ*_em_ at 475 and 520 nm) and PL spectrum (*λ*_ex_ at 400 nm excitation) of His-AuNCs in the solid state. (b) Low resolution TEM image of His-AuNCs. Inset – HRTEM image.

The PL emission of the lyophilized His-AuNCs was investigated *via* steady-state fluorescence spectroscopy. Fig. S1† displays the emission spectra of the solid-state-His-AuNCs at different excitation wavelengths, starting at 340 nm up until 500 nm. The powder shows two wide emission bands at 475 and 520 nm that cover the dominant part of the visible spectra (400–700 nm). Interestingly, the ratio between peak intensities changes with the excitation wavelength. For example, the higher energy emission (475 nm) presents a dominant intensity under excitation between 340 and 400 nm, while under 420 nm excitation or above, the lower energy band takes over. We have previously observed in our studies an excitation-dependent emission for AuNCs.^21^ Even though, to our knowledge, there is no concrete theory that effectively explains the intrinsic PL of AuNCs, we believe that the PL band at around 475 nm could be attributed to the Au core, according to the metal confinement effect, while the band at approximately 520 nm might originate from the surface-attached His, as it was demonstrated that the PL band of His-AuNCs displays a gradual bathochromic shift with the increase in the number of capped ligands,^22^ which could occur in our case during the lyophilization process. Furthermore, we evaluated the excitation spectra of the two emission bands and the results are presented in Fig. 1a. The 475 nm emission band exhibits an excitation shoulder located at 380 nm, while the 520 nm one displays a well-defined excitation peak centered at 440 nm, which are in excellent correlation with the aforementioned results. Regarding UV-vis absorption properties, we have shown in our previous publication^20^ that His-AuNCs exhibit two weak absorption bands at 256 and 324 nm, related to the presence of stabilizing His. The absence of a specific surface plasmon resonance band at around 520 nm indicates that the synthesized nanomaterial has nanocluster dimensions, which was further confirmed by TEM.

The representative overall image presented in Fig. 1b and the high resolution one (inset – Fig. 1b) highlight the presence of well-defined individual very small spherical particles. After analyzing more than 100 particles, the average size of the His-AuNCs was calculated to be 2.9 ± 0.3 nm.

### 3.2 Structural characterization

**Fig. 2:**
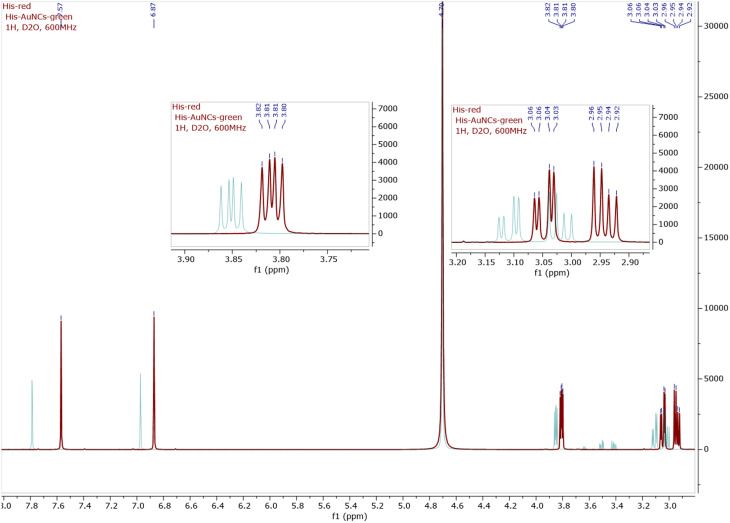
The ^1^H-NMR superimposed spectra of His (red) and His-AuNCs (green), including the detailed view of the aliphatic region. The ^1^H-NMR of both His and His-AuNCs were recorded at 600 MHz in D_2_O.

A structural characterization of His-AuNCs was first made based on NMR spectroscopy (^1^H NMR, ^13^C NMR, and COSY, see Fig. 2 and ESI Fig. S2–S8†). As presented in the ^1^H-NMR spectra of His (red line) and His-AuNCs (green line), all the signals corresponding to the His protons in His-AuNCs are significantly deshielded compared to those of His (Fig. 2).

The most deshielded signal of His-AuNCs, with the chemical shift *δ* = 7.78 ppm, belongs to a proton situated between the nitrogen atoms of the 1H-imidazole ring (see in Fig. S2† the structure of His and the numbering used to assign the signals), with the corresponding signal of His having a chemical shift at *δ* = 7.56 ppm. The highest difference between the chemical shifts of the protons from His-AuNCs compared to those from His was 0.22 ppm for the proton Ha (*δ*_Ha_ = *δ*_(Ha)His-AuNCs_ − *δ*_(Ha)His_), followed by proton Hb (*δ*_Hb_ = 0.1 ppm) and diastereotopic protons Hc (*δ*_Hc_ = 0.06 ppm; *δ*_Hc’_ = 0.07 ppm) and Hd (0.05 ppm). The incidence of the proton signals at a lower magnetic field in His-AuNCs is a result of the His coordination on the Au surface, which affect the electron density in the hole molecule, being more pronounced on the 1H-imidazole ring, but remain significant even for aliphatic protons.

**Fig. 3:**
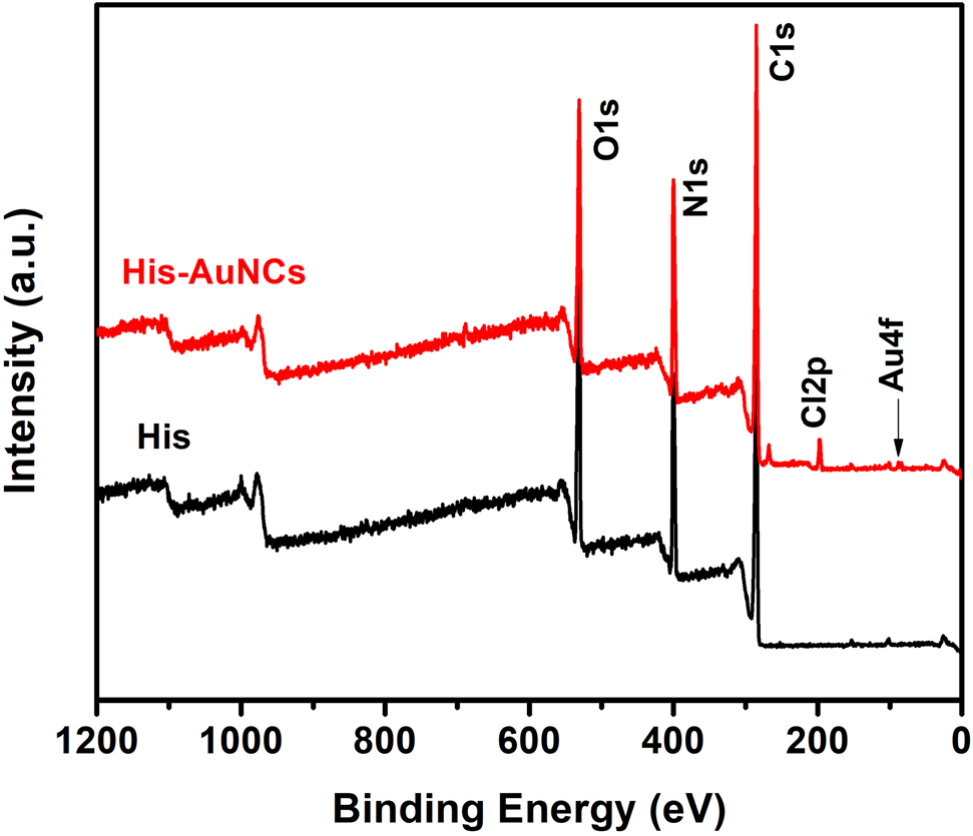
XPS survey spectra of His and His-AuNC samples.

Furthermore, to obtain information on the elemental composition of His-AuNCs and to better understand how AuNCs interact with His, XPS analysis was performed. The survey XPS spectra of the His and His-AuNCs samples are shown in Fig. 3.

The photoelectron peaks corresponding to C, N, O and Au, respectively, can be clearly identified in the survey spectra. XPS spectra in the regions of C 1s, O 1s, N 1s and Au 4f core levels together with the peak-fitting results are shown in Fig. S9–S12.† A careful analysis of the survey and high-resolution spectra could reveal important information about the chemical composition of the samples and also about the oxidation state of Au in the His-AuNC sample in order to verify the type of chemical bond which forms between Au and His. Under suitable conditions, the three functional groups, amino, imidazole (IM) and carboxylic acid of His are potential binding sites to form a complex with the metal ions.^23^

The elemental composition on the surface of His and His-AuNC samples obtained by the analysis of XPS scans in Fig. 3 is summarized in Table S1.† Comparing the atomic concentrations for the two samples, one can notice a slight decrease in the weight of oxygen and carbon for the His-AuNC sample compared to the His sample. At the same time, a significant increase in nitrogen is observed. The Au atomic concentration is very low, being around 0.1% in the layer analyzed by the XPS technique, which is up to 10 nm. In addition, the presence of chlorine was also observed in the His-AuNC sample.

The high-resolution C 1s spectra recorded for the two samples have an asymmetric shape. The width of the peaks allowed a deconvolution using four components (Fig. S9†), which are summarized in Table S2.† At lower binding energies, of around 284.6 eV, there is a component attributed to the carbon atom involved in C–C/C–H bonds. This component was used as a reference for the calibration of all recorded XPS spectra. The second component at 285.8 eV can be due to the C–N bonds in His and/or C–O coming from the contaminating carbon due to the atmospheric exposure of the samples. The component at around 288 eV is attributed to the carbon in the carbonyl groups (C=O).^23^ The peak of carbon in carboxylate (O=C–OH), amide carbon (N–C=O) and/or imidazole's N=C–NH bond of His appeared at 289.9 eV in the His sample whereas in the His-AuNC C 1s spectrum, this peak appeared at 290.3 eV with a shift of 0.4 eV to a higher binding energy when Au bonds to His.^24^

It was shown that the imidazole's N=C–NH carbon peak is located at a binding energy of 290.5 eV, whereas the amine's carbon (C–NH_2_) peak is positioned at 290.0 eV.^25^ The shift to higher binding energies of this component peak for the His-AuNC sample indicates a slightly increased contribution of the imidazole carbon connected with protonated nitrogen.

The high-resolution N 1s spectra and their deconvolutions for the His and His-AuNC samples are shown in Fig. S10.† Both spectra were well fitted using two spectral components each. The N 1s spectrum for the His sample has two component peaks at 398.8 eV and 400.7 eV, while for the His-AuNC sample the peaks are located at 398.6 eV and 400.5 eV. The results obtained from the deconvolutions are centralized in Table S3.† For the His-AuNC sample it was noted that there is a shift of the N 1s spectrum components by 0.2 eV towards lower values of the binding energies accompanied by a change in the weight of the two components compared to in the spectrum of the His sample. The component at lower binding energy could be assigned to an imino-nitrogen atom (IM-ring) and those at higher values are related to the C–NH–C bond in an IM-ring and to nitrogen atoms of the amino group (NH_2_) of His.^23^ The weight of the N 1s component at lower binding energy decreases after His–Au interaction and an increased amount of the amino spectral component is observable. His molecules bond to each other by strong intermolecular forces. It is known that His molecules are predominantly zwitterionic.^26^ Two types of zwitterions with protonated amino groups and with protonated imidazole rings, could be present in His samples. The N 1s spectrum of the analyzed samples lacks the component corresponding to protonated amines (NH^3+^) which would appear at around 402 eV.^26^ Thus, when gold bonds to His, a partial protonation of the nitrogen in the imidazole cycle is very likely because the component at 398.6 eV assigned to the imino-nitrogen atom (IM-ring) decreases in weight and the one at 400.5 eV increases.

The high-resolution O 1s spectra are shown in Fig. S11.† The spectrum of the His sample could be decomposed into four peaks associated with O–C=O, C=O or O=C–N (∼531 eV), C–OH, O̲=C–O (∼532.4 eV) and O=C–O̲, C–O–C (∼533.8 eV) bonds,^24^ as summarized in Table S4.† The same four components, with the same assignment, were used for the deconvolution of the O 1s spectrum for His-AuNCs. The peaks were located at 530.8 eV, 532.2 eV and 533.9 eV, respectively.

The observation of the protonated amino group in the IM ring together with the dominant O 1s component at around 531 eV indicates a proton transfer from the carboxyl group of His to the amino group, and the formation of a zwitterionic His molecule. On analyzing the above presented results, it can be considered that the shifting of carboxylate and amino peaks of His for the His-AuNC sample compared to for the His sample indicates surface interaction of Au and His through carboxyl and amino groups. Thus, binding is likely to occur through covalent (Au with His molecules through IM-ring amino functionalities) and electrostatic interactions (with carboxyl). A stabilization of the non-protonated state of the amino group and the protonated nitrogen atom of the imidazole ring may be achieved by coordination to gold atoms. Thus, active N-sites contribute to the His substrate bonding, in addition to the carboxyl groups.^26^

The spin–orbit splitting doublet peaks of the Au 4f core-level spectrum for the His–Au sample, (Au 4f_5/2_ and Au 4f_7/2_ with a peak to-peak distance of 3.7 eV) (see Fig. S12†) are of symmetrical shapes. The Au 4f_7/2_ peak is located at 84.4 eV and corresponds to Au(i) of the surface bonding species. The spectra showed no obvious overlap of the contributions related to the Au(0) species which should be located at a binding energy of 83.9 eV for Au 4f_7/2_.^26^ The deconvolution of the Au 4f spectrum allowed the precise identification of the position of the two components Au 4f_7/2_ and Au 4f_5/2_ at 84.5 eV and 88.2 eV, respectively (Table S5†). The existence of Au(i) species suggests the formation of a His–Au complex (nanoclusters).^27^ The presence of Au(i) could be closely related to the luminescence of the sample.^28^ The presence of Au(i) clearly indicates that the gold atoms at the surface acquire a partial positive charge at the interface with the His molecules. His is probably an anionic form that creates a strong ionic-covalent bond with Au.

**Fig. 4:**
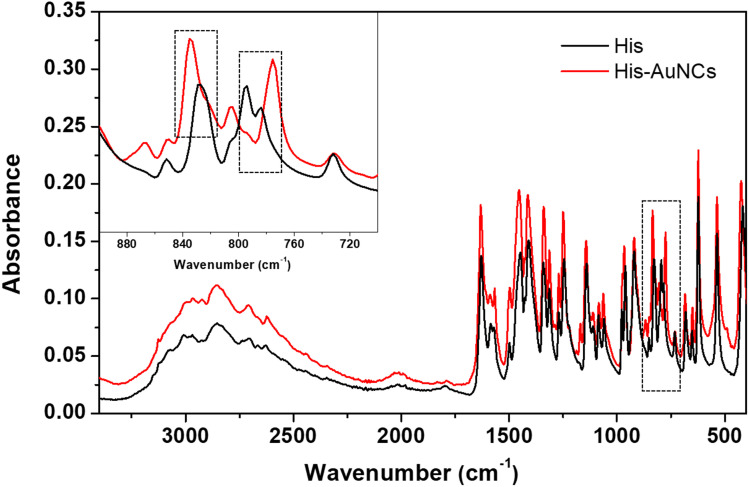
The FT-IR spectra of His (black) and His-AuNCs (red). The inset displays the extended spectra in the 700–900 cm^−1^ region.

Additionally, in order to strengthen our findings on the interaction of His with AuNCs, we also performed FT-IR spectroscopy measurements. Fig. 4 displays the FT-IR spectra of free His and His-AuNC samples. The two spectra look very similar, however, on closer observation, there is a subtle difference in the 750–850 cm^−1^ region. Specifically, the bands at 785 cm^−1^ and 828 cm^−1^ are shifted to 775 cm^−1^ and 835 cm^−1^, respectively, for His-AuNCs compared to free His. According to the literature, these two bands are attributed to the CH_2_ (C_2_) rocking motions and C_1_–C_2_ stretches along the backbone of His, respectively. Therefore, the modification of their shape, position and relative intensity indicates that His binds to AuNCs through the carboxyl and amino moieties, as previously demonstrated by XPS measurements.

Finally, mass spectrometry measurements confirmed the presence of stabilizing His on the synthesized AuNCs. The mass spectra presented in Fig. S13† demonstrate that the fragmentation pathway from the His-AuNC sample is characteristic of that obtained from His since the fragmentation peaks with *m*/*z* 110, 93, and 81 are present for both free His and His-AuNCs.

### 3.3 Fluorescence lifetime imaging microscopy assays

**Fig. 5:**
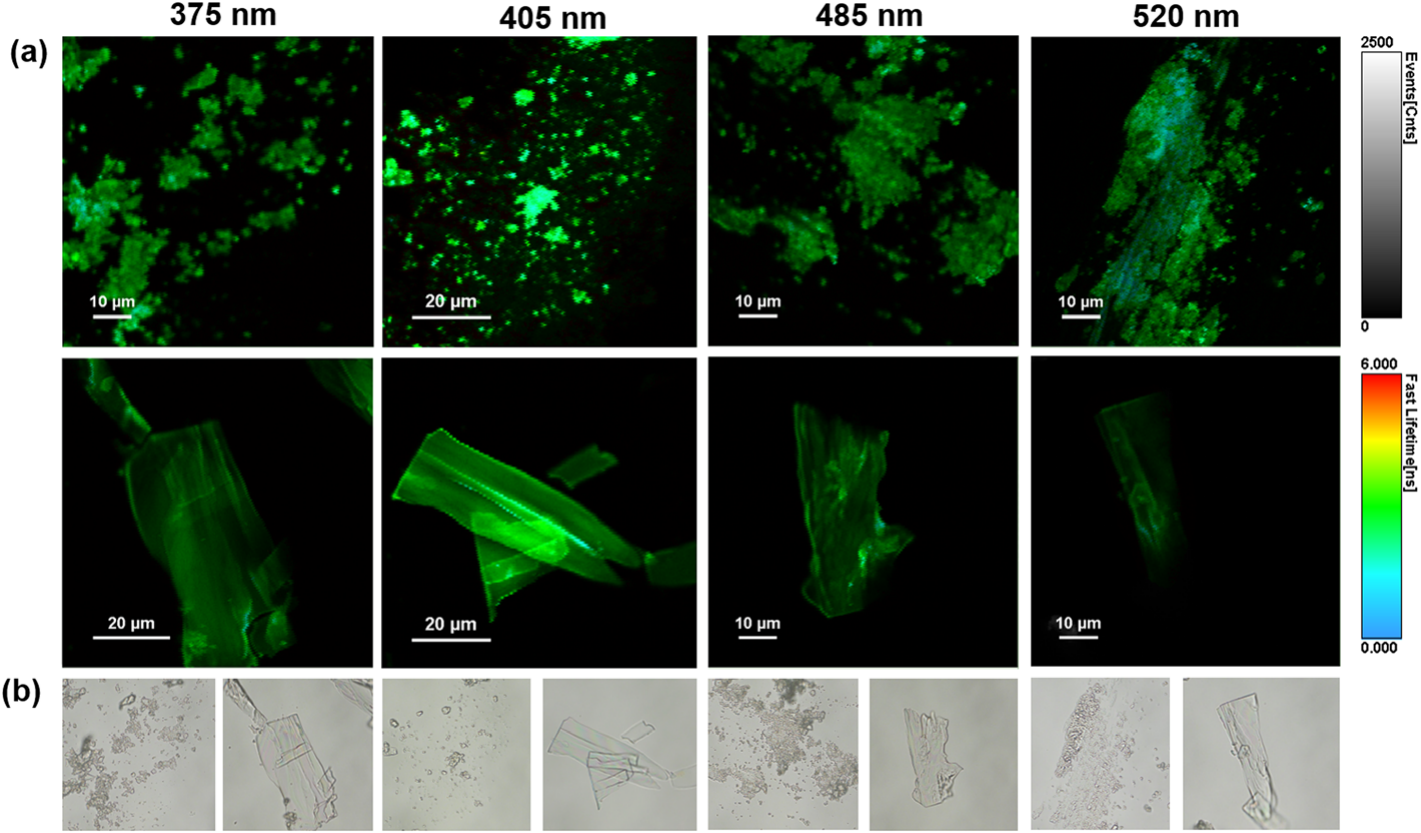
(a) FLIM images of powder His-AuNCs deposited on a glass substrate, obtained at different excitation wavelengths (375, 405, 485 and 520 nm). (b) Corresponding optical images of the powder areas investigated in the FLIM images presented in (a).

Subsequently, the fluorescence emission of His-AuNC powder was investigated *via* fluorescence lifetime imaging microscopy (FLIM) at multiple excitation wavelengths (375, 405, 485 and 520 nm). FLIM images obtained from selected areas are presented in Fig. 5a together with their corresponding optical images (Fig. 5b).

Under all excitations, the PL of the His-AuNCs presents a uniformly distributed emission throughout the flakes. The best PL-performance was obtained under 405 nm excitation which is correlated with the results obtained by conventional fluorescence spectroscopy (Fig. S1†). Moreover, the lifetime decay curves were extracted from different parts of the flakes (Fig. S14†). The average lifetimes obtained after fitting the decay curves are presented in Table S6.† The flakes exhibit fast average-lifetimes ranging between 2 and 3 ns. Interestingly, under 520 nm excitation, the emission lifetime of His-AuNCs decreases to 2.15 ns.

Under this excitation, the emission from 475 nm presents low to no contribution to the average lifetime, suggesting that the lower energy emission, localized at 520 nm, exhibits faster lifetimes compared to the higher energy one, from 475 nm. These results are correlated with the FLIM images (Fig. 5a) where light blue spots (faster lifetimes) were observed under 520 nm excitation.

### 3.4 White-light generation and photo-thermal resistance

**Fig. 6:**
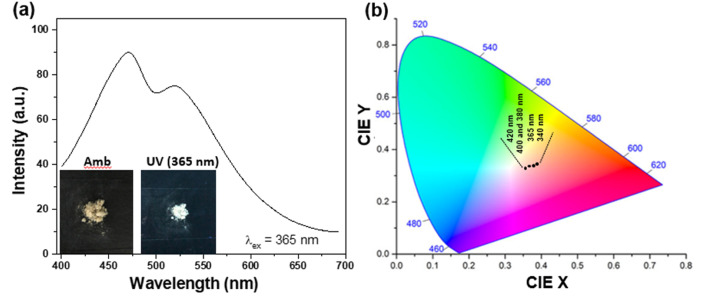
(a) PL spectrum of powder His-AuNCs under excitation at 365 nm. Inset – photographic image of His-AuNCs in the solid state under ambient and UV light exposure (365 nm) proving white-light generation. (b) The CIE color coordinates of His-AuNCs in the solid state under different excitation wavelengths in the 340–420 nm range.

In order to be truly promising towards practical applications, a WLEM should be able to produce white-light in solid- or gel-states. Solid-state His-AuNCs present a dark-yellow color in ambient light. However, when the powder is irradiated with a UV lamp, it turns white (Fig. 6a – inset). The emission spectrum, obtained under 365 nm excitation, exhibits two emission bands (470 and 520 nm) which extend over the visible spectrum, in the 400–700 nm region (Fig. 6a). Furthermore, for a deeper chromatic interpretation, the CIE chromaticity diagram was used to evaluate the white-purity of the light emitted under different excitation wavelengths (Fig. 6b). The coordinates obtained at different excitation wavelengths are presented in Table S7.† For example, the CIE coordinates of the solid-state His-AuNC emission under 420 nm excitation were (0.36, 0.33), extremely close to the values of a perfect white-light emitting source which are (0.33, 0.33)^6^ and comparable, for example, with the result obtained for more popular dibenzothiophene-based phosphors which were reported to exhibit white light with CIE coordinates up to (0.33, 0.35).^29^ On the other hand, His-AuNCs reveal a superior performance compared to reported WLEMs based on doped organic gels exhibiting white-light emission with (0.28, 0.34) CIE coordinates^30^ or some of the WLEMs based on metal-halide perovskites.^31^ Nevertheless, as proved by the obtained CIE coordinates, solid-state His-AuNCs are capable of generating a nearly white-light under excitations ranging between 365 and 420 nm, demonstrating their appealing emitting properties.

**Fig. 7:**
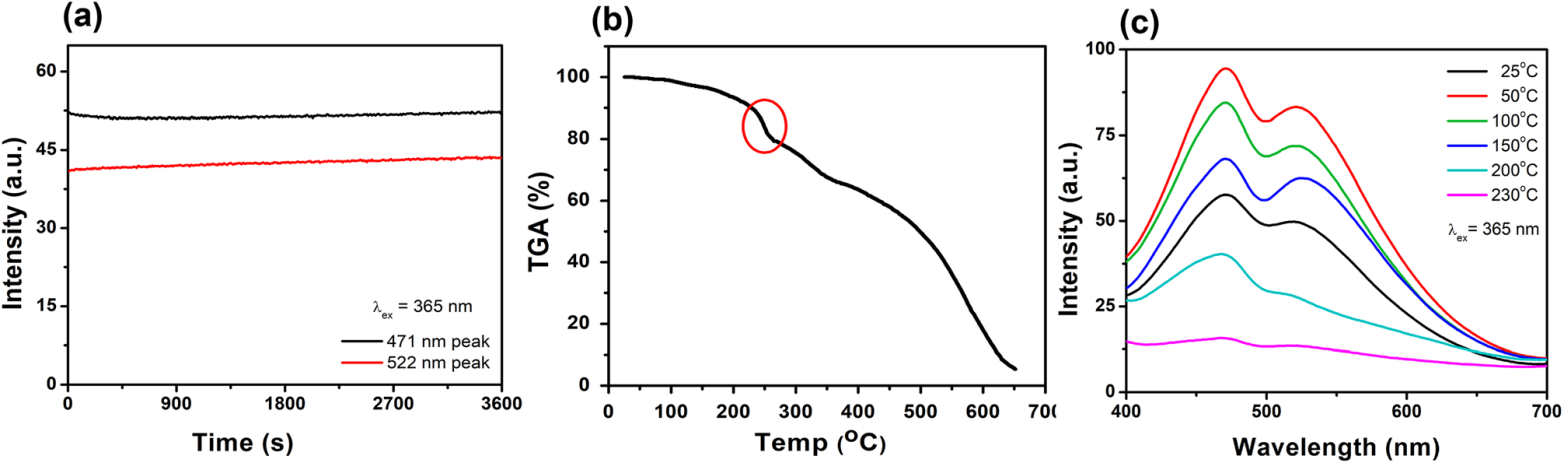
(a) PL intensity under continuous excitation at 365 nm. (b) Thermogravimetric analysis (TGA) curve. (c) PL spectra of powder His-AuNCs at 365 nm excitation, after thermal treatment at various temperatures in the 25–230 °C range.

Moreover, for a WLEM, a great emission intensity and an excellent CIE coordinate range are not enough to be considered as a reliable and efficient white-light emission source. Thus, photostability and thermic stability tests were performed on the His-AuNC powder. After being irradiated for 1 h at a 365 nm excitation wavelength (Fig. 7a), the solid-state His-AuNCs exhibit great photostability for both emission peaks. Another important feature of a WLEM is the thermal decomposition level, or so to say, at what temperature it loses the ability to emit white-light. Therefore, thermogravimetric analysis (TGA) was performed on the His-AuNC powder. The TGA curve (Fig. 7b) presents two important weight loss steps. The first weight loss occurs at 110–180 °C, which corresponds to the evaporation of crystallized water, representing a small fraction. The main weight loss occurs with the decomposition of the His-AuNC complex at 220–280 °C (red mark in Fig. 7b). The His and the Au0 bonds break at the given temperature, and a high weight loss occurs. The two emission bands are well preserved until 150 °C but when the temperature exceeds 200 °C the solid-state His-AuNCs start to lose their PL properties (Fig. 7c).

The CIE color coordinates were calculated for the given temperatures in Table S8,† and it can be seen that the His-AuNC powder maintains its WLEM properties up until almost 200 °C, a relatively high temperature. The fact that they are not warming up under UV irradiation, represents an advantage compared to commercially available WLEDs which are continuously dissipating heat during use. Additionally, other WLEMs based on NCs are in general multi-component mixtures^6,32^ while the one proposed in our work is a single-component emitting material, making it easier to fabricate and evaluate.

Therefore, solid-state His-AuNCs represent a cost-efficient, eco-friendly, photo-stable and thermo-stable WLEM that shows great promise to be implemented in future manufacturing of WLED.

## 4. Conclusions

In conclusion, we successfully fabricated a novel, reliable, eco-friendly, cheap and health-friendly WLEM based on His-AuNCs in solid state. The employed photoluminescent material was obtained through the lyophilization of His-AuNCs with an average size of 2.9 ± 0.3 nm, synthesized *via* a microwave-assisted procedure. The obtained powder exhibits a broad dual-band emission (475 and 520 nm) expanding over an important area of the visible spectrum ensuring white-light emission under UV excitation. The excellent purity of the generated white-light was confirmed by the coordinates obtained using the CIE chromaticity diagram, at different wavelengths. Finally, their high photo- and thermo-stability prove their ability to perform as a reliable, eco-friendly, cheap and energy-efficient WLEM for the development of rare-earth-free material-based WLEDs.

## Author contributions

M. Z. and A. M. H. contributed equally to this work. Conceptualization: A. M. C. and A. M. H.; methodology: A. M. H. and M. Z.; validation: A. M. H., M. Z., M. T., and L. I. G.; investigation: A. M. C., A. M. H., M. Z., M. T., and L. I. G.; writing – original draft preparation: A. M. H. and M. Z.; writing – review and editing: A. M. C., and S. A.; supervision: A. M. C. and S. A.; funding acquisition: A. M. C. All authors have read and agreed to the published version of the manuscript.

## Conflicts of interest

There are no conflicts to declare.

## Supplementary Material

NA-005-D3NA00555K-s001
