# Supplementary material for: Dual-emissive solid-state histidine-stabilized gold nanoclusters for applications in white-light generation
Source: Nanoscale Adv. 2023 Sep 14;5(21):5810–8. doi: 10.1039/d3na00555k (PMC10597560; doi:10.1039/d3na00555k)
Supplement: NA-005-D3NA00555K-s001 [file NA-005-D3NA00555K-s001.pdf]

Electronic Supplementary Material (ESI) for Nanoscale Advances.
This journal is © The Royal Society of Chemistry 2023

Electronic Supplementary Information

Dual-emissive solid-state histidine-stabilized gold nanoclusters for applications in white-light generation

Markus Zetes[a,b,#], Alexandru-Milentie Hada[a,b,#], Milica Todea[c,d], Luiza Ioana Gaina[e], Simion Astilean[a,b], Ana-Maria Craciun[a,*]

[a] Nanobiophotonics and Laser Microspectroscopy Center, Interdisciplinary Research Institute in Bio-Nano-Sciences, Babes-Bolyai University, 42 T. Laurian Str., 400271, Cluj-Napoca, Romania

[b] Faculty of Physics, Babes-Bolyai University, 1 M. Kogalniceanu str., 400084, Cluj-Napoca, Romania

[c] Nanostructured Materials and Bio-Nano-Interfaces Center, Interdisciplinary Research Institute in Bio-Nano-Sciences, Babes-Bolyai University, 42 T. Laurian Str., 400271, Cluj-Napoca, Romania

[d] Department of Molecular Sciences, Faculty of Medicine, Iuliu Haţieganu University of Medicine and Pharmacy, Cluj-Napoca, Romania

[e] Research Center on Fundamental and Applied Heterochemistry, Faculty of Chemistry and Chemical Engineering, Babes-Bolyai University, 11 A. Janos Str., 400028, Cluj-Napoca, Romania

**Corresponding author*: ana.gabudean@ubbcluj.ro

[#] *These authors contributed equally to this work*

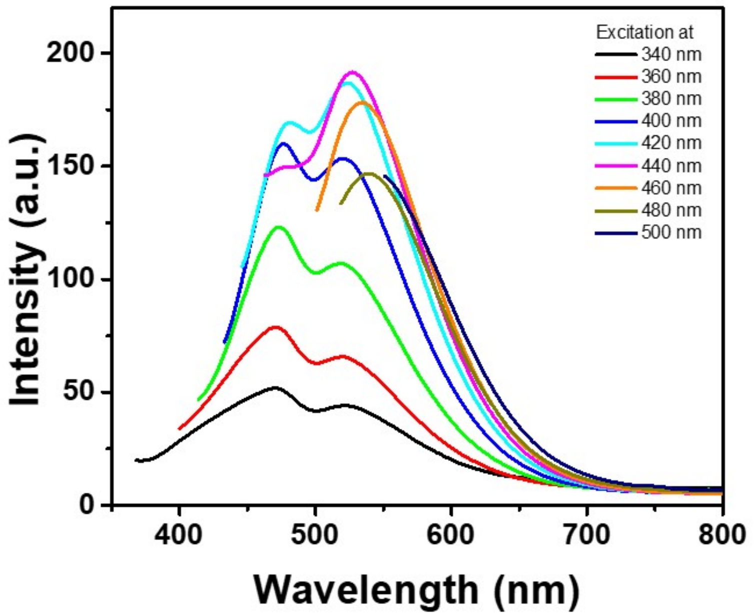

Figure S1. PL spectra of powder His-AuNCs at different excitation wavelengths.

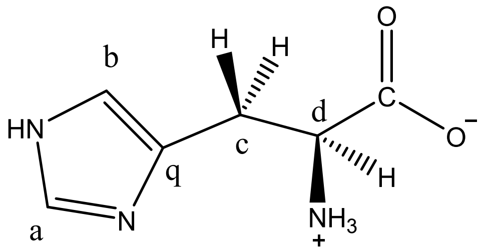

Figure S2. The L-histidine structure with the imidazole ring and the numbering used to assign the signals in ^{1}H and ^{13}C NMR spectra.

L-Histidine

^{1}H-NMR (600MHz, D_2O, δppm): 7. 56 (s, 1H, Ha), 6.87(s, 1H, Hb), 3,80(dd, 1H, J=8.01Hz, J=4.75Hz, Hd), 3.04(dd, 1H, J=0.74Hz, J=15.4Hz Hc'), 2.94(dd, 1H, J= 8.01Hz, J=15.4Hz, Hc)

^{13}C-NMR (150MHz, D_2O, δppm): 28.1Cc, 54.7Cd, 116.6Cb, 132.1Cq, 136.2Ca, 173.9 Cq(C=)O

His-AuNCs

^{1}H-NMR (600MHz, D_2O, δppm): 7.78(s, 1H, Ha), 6.97(s, 1H, Hb), 3.85(dd, 1H, J=7.8 Hz, J=5.0 Hz, Hd), 3.10(dd, 1H, J= 15.5Hz, J= 5.0Hz, Hc') 3.01(dd, 1H, J=15.5 Hz, J=7.8 Hz, Hc)

^{13}C-NMR (150MHz, D_2O, δppm): 27.6 Cc, 54.5 Cd, 116.8 Cb, 131.2 Cq, 135.8 Ca, 173.5 Cq(C=)O

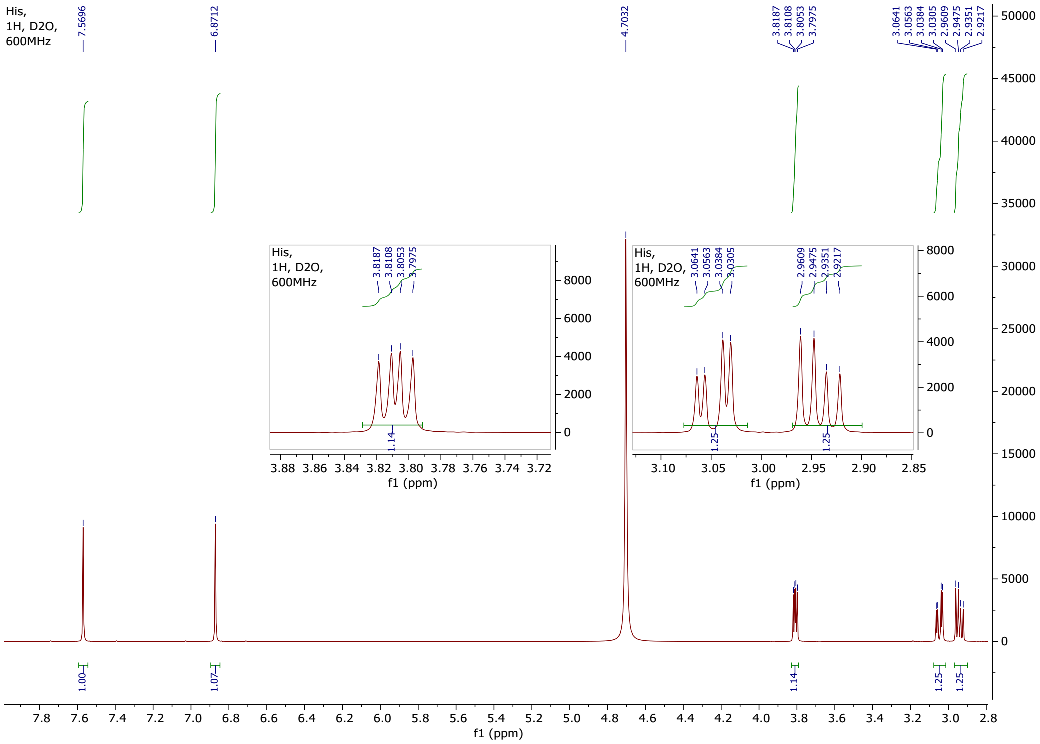

Figure S3. ^{1}H-NMR spectra of L-histidine at 600 MHz in D_2O, including the detail of aliphatic region.

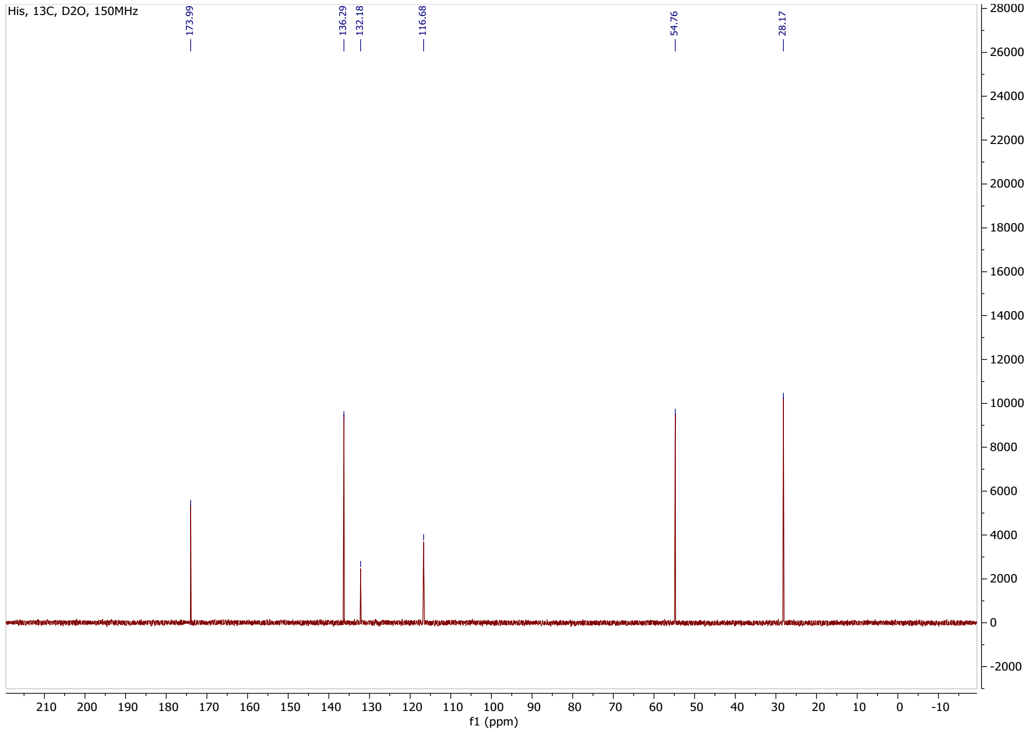

Figure S4. ^{13}C-NMR spectra of L-histidine at 150 MHz in D_2O.

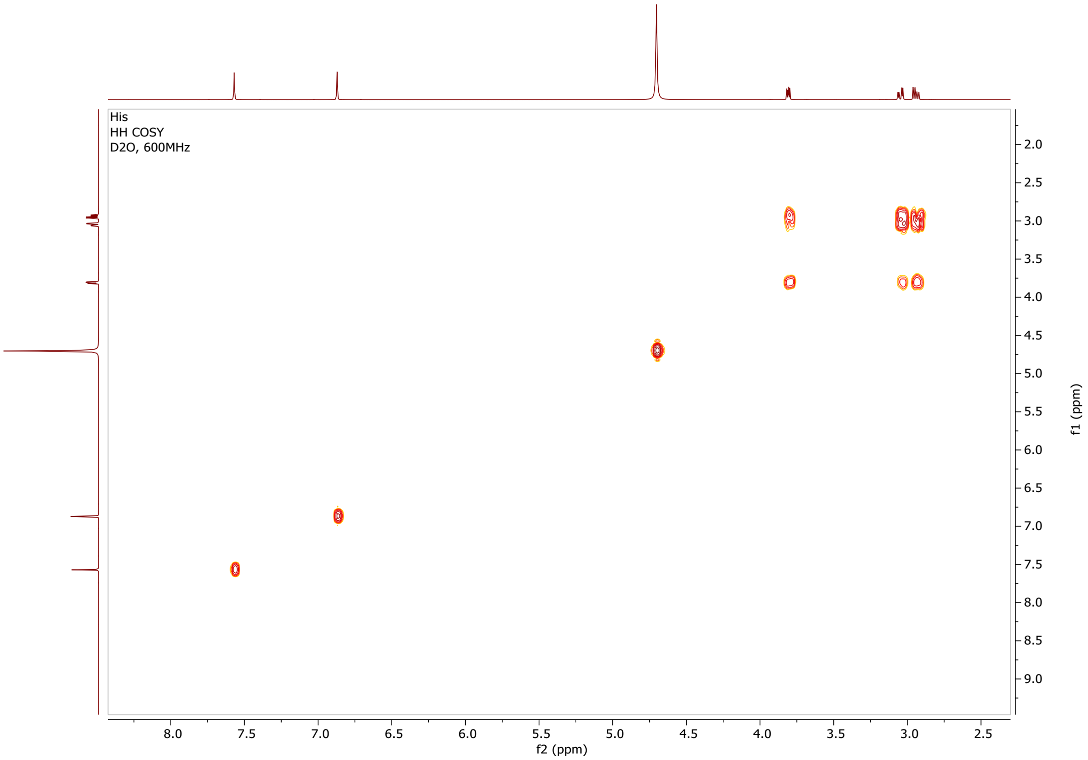

Figure S5. The ^1H-^1H COSY spectra of L-histidine.

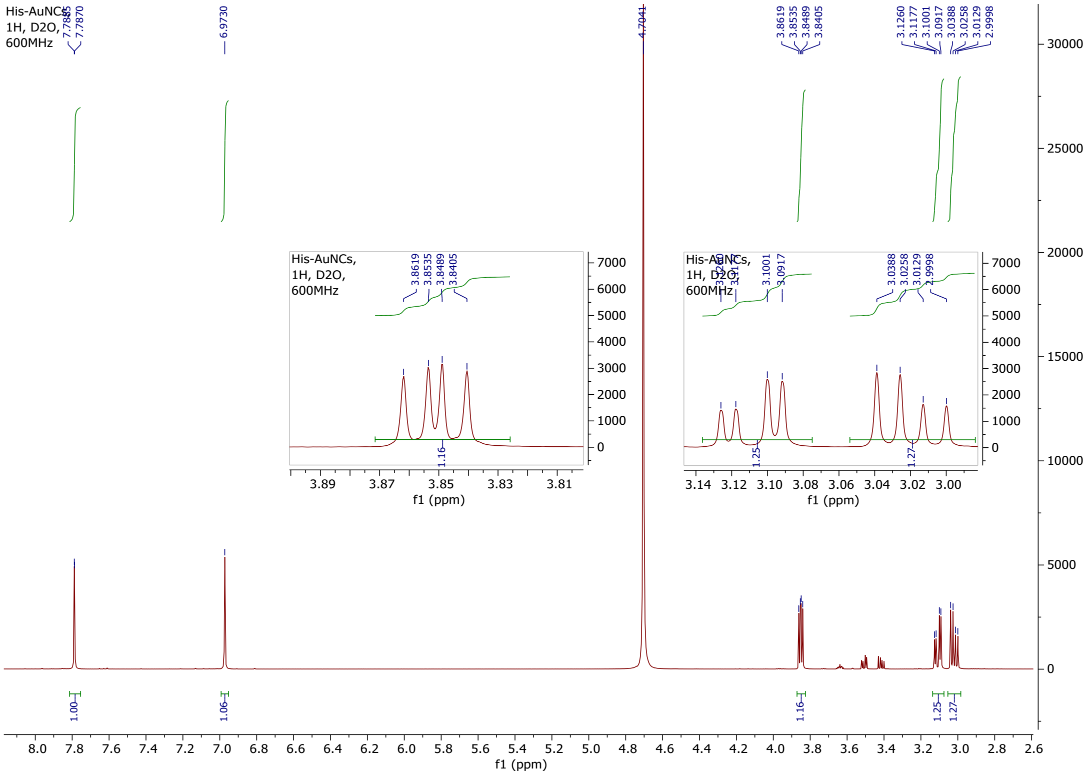

Figure S6. ^{1}H-NMR spectra of His-AuNCs at 600 MHz in D_2O, including the detail of aliphatic region.

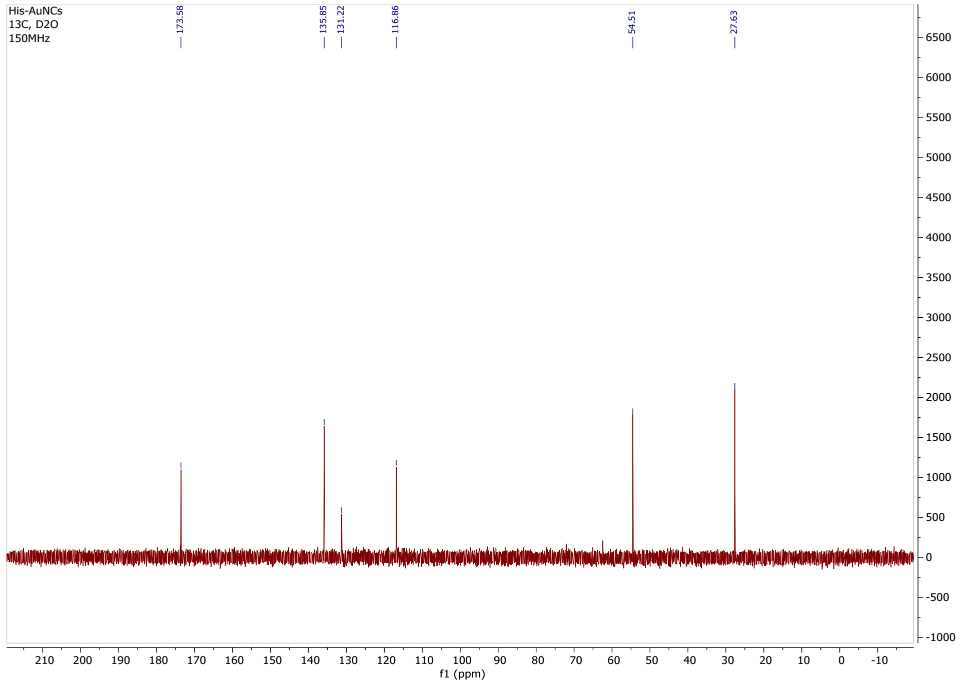

Figure S7. ^{13}C-NMR spectra of His-AuNCs at 150 MHz in D_2O.

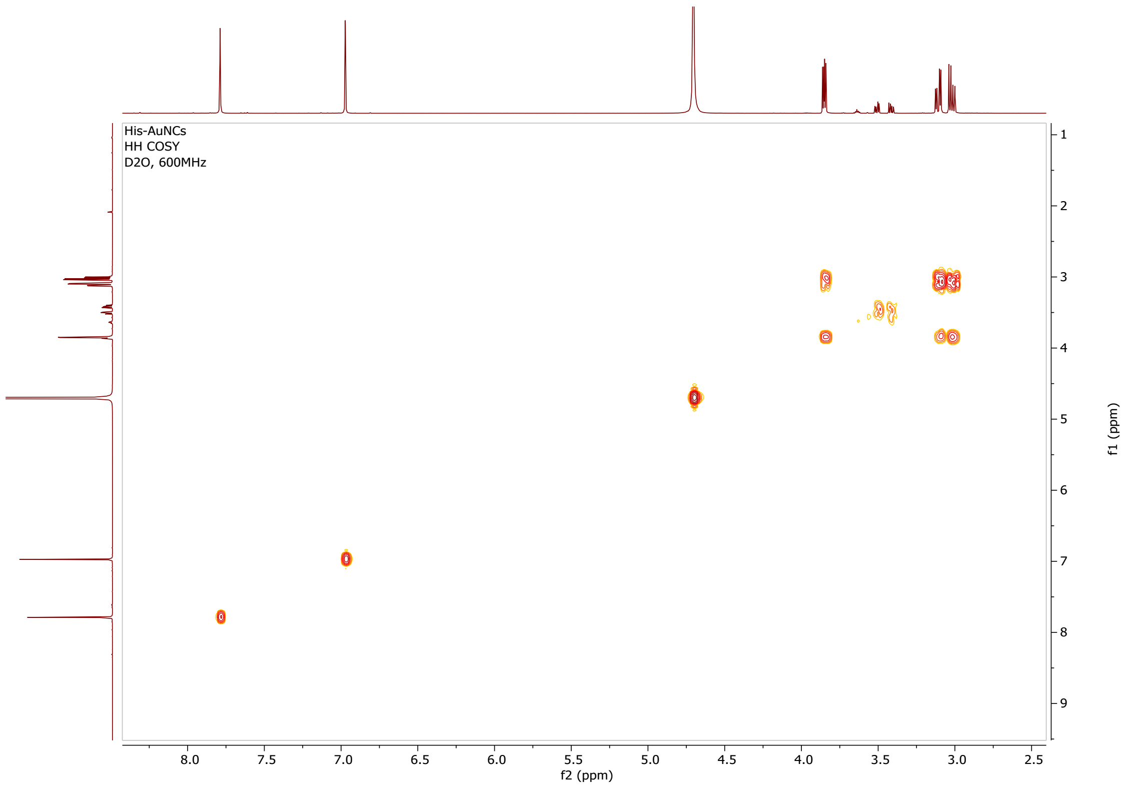

Figure S8. The ^{1}H-^{1}H COSY spectra of His-AuNCs.

Table S1. Elemental at% composition at the surface of the His and His-AuNCs samples.

Sample	at%				
	**O**	**C**	**N**	**Cl**	**Au**
**His-AuNCs**	15.1	62.4	20.3	2.1	0.1
**His**	17.6	64.5	17.9	-	-

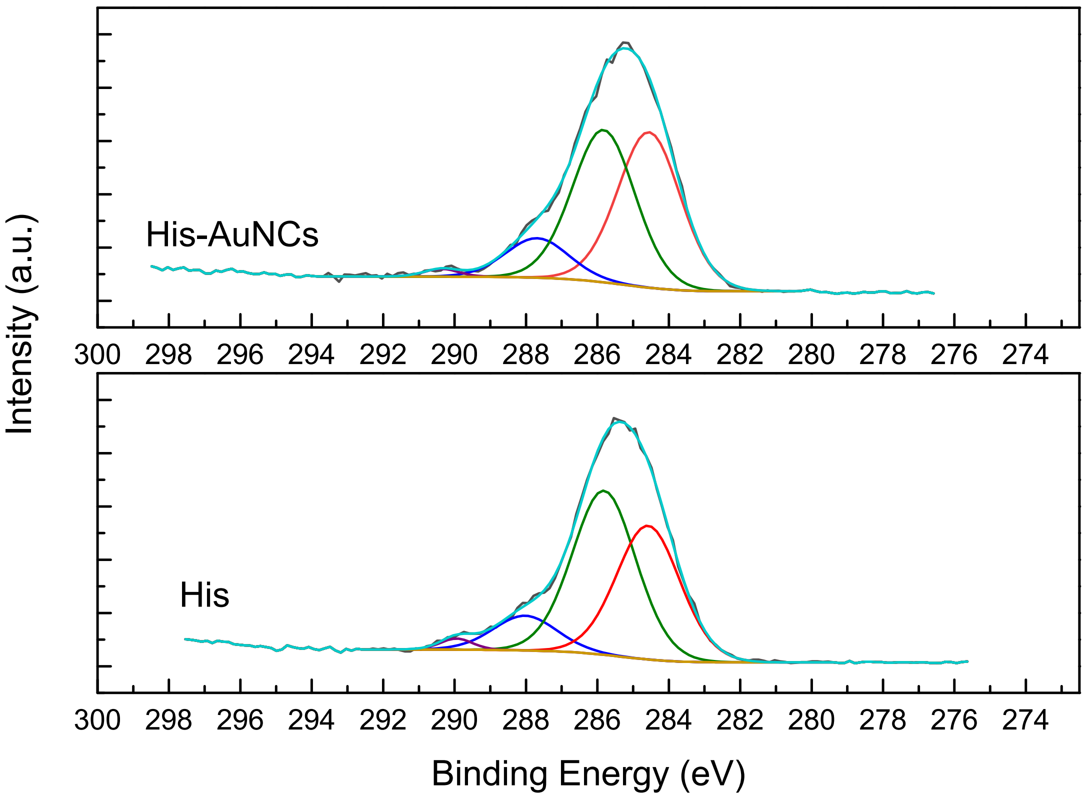

Figure S9. C1s core level spectra.

Table S2. Deconvolution of C1s spectra.

Sample/component	C-C<br>C-H	%	C-N<br>C-O	%	C=O	%	O=C–OH<br>N–C=O	%
**His-AuNCs**	284.6	44.2	285.8	43.3	287.7	11.3	290.3	1.2
**His**	284.6	39.5	285.8	48.5	288	10.3	289.9	1.7

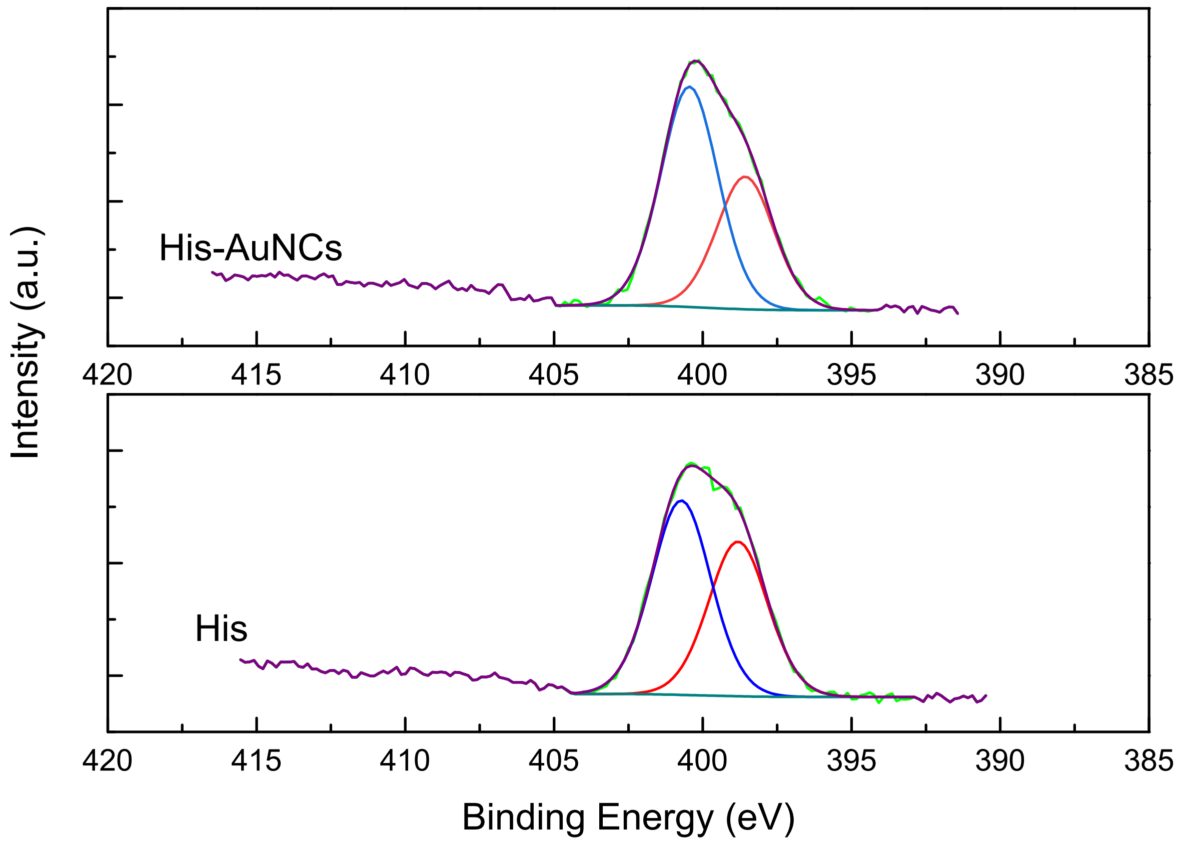

Figure S10. N1s core level spectra.

Table S3. Deconvolution of N1s spectra.

**Sample/component**	**Imino N in IM ring**	**%**	**(-NH2) amino group C-NH-C (IM-ring)**	**%**
**His-AuNCs**	398.6	37.6	400.5	62.4
**His**	398.8	44.3	400.7	55.7

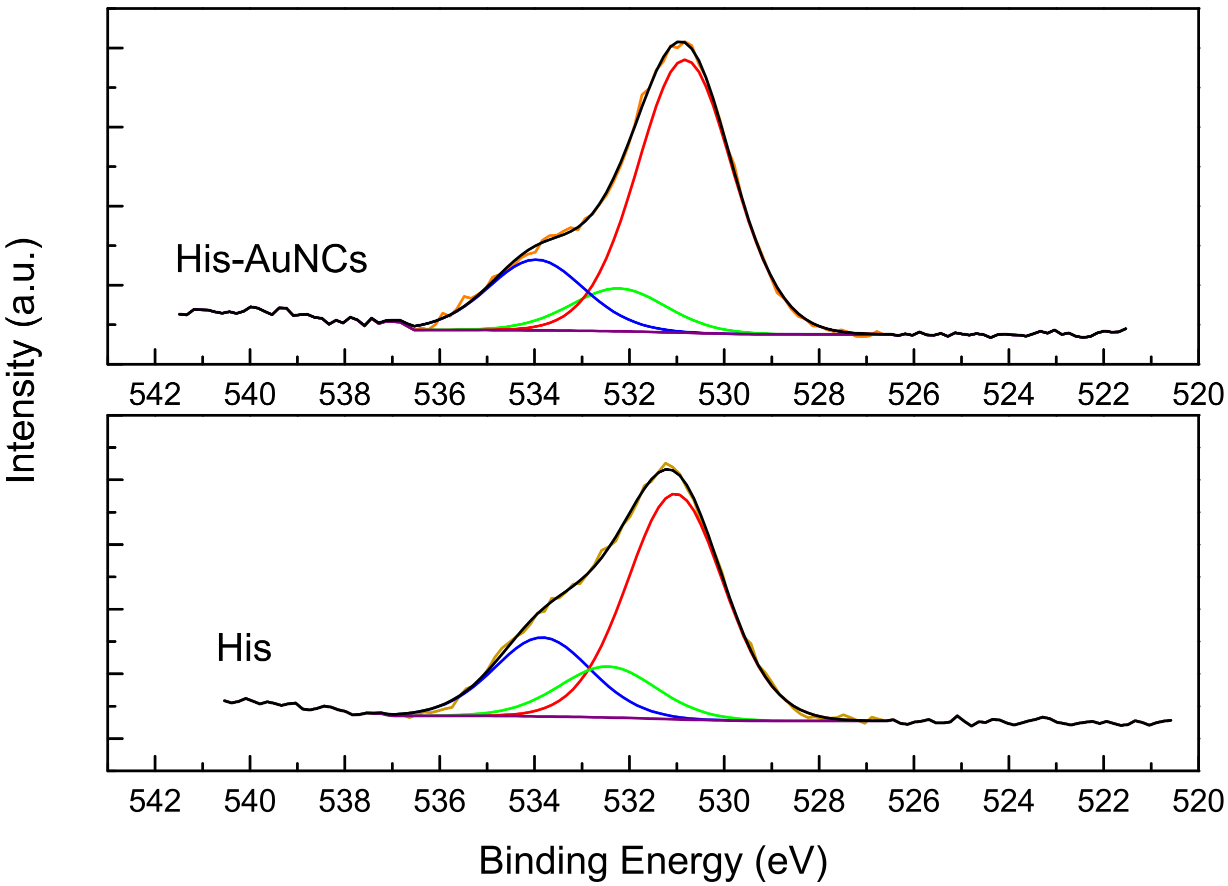

Figure S11. O1s core level spectra.

Table S4. Deconvolution of O1s spectra

**Sample/component**	**HO-C=O<br>O=C-N<br>C=O**	**%**	**C-OH<br>O=C-O**	**%**	**O=C-O<br>C-O-C**	**%**
**His-AuNCs**	530.8	70.6	532.2	11.1	533.9	18.3
**His**	531	63.4	532.4	14.4	533.8	22.2

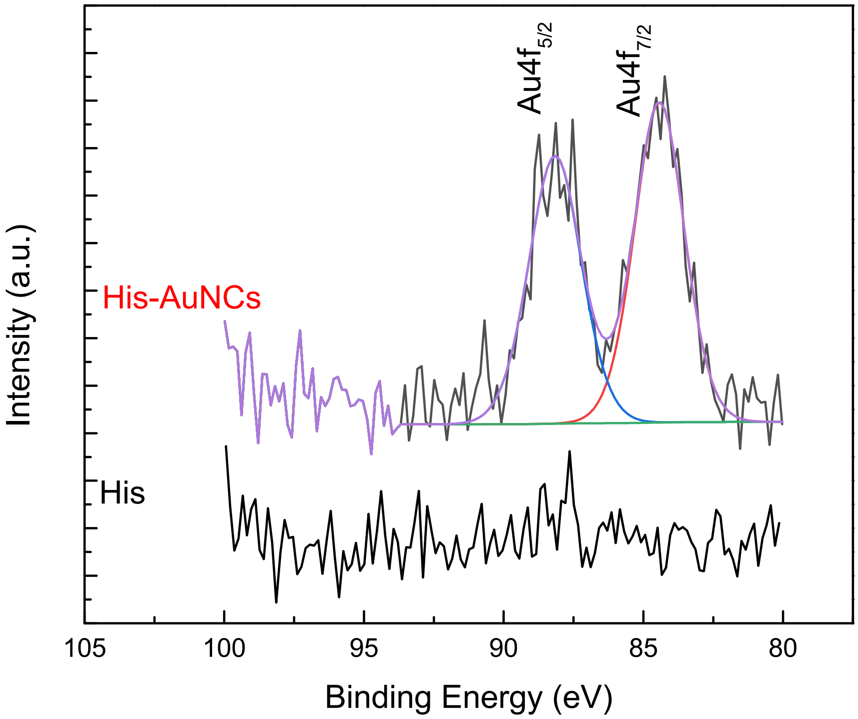

Figure S12. Au4f core level spectra.

Table S5. deconvolution of Au4f spectra

Sample/component	$Au4f_{7/2}$	%	$Au4f_{5/2}$	%
**His-AuNCs**	84.5	57.1	88.2	42.9
**His**	-	-	-	-

(a)

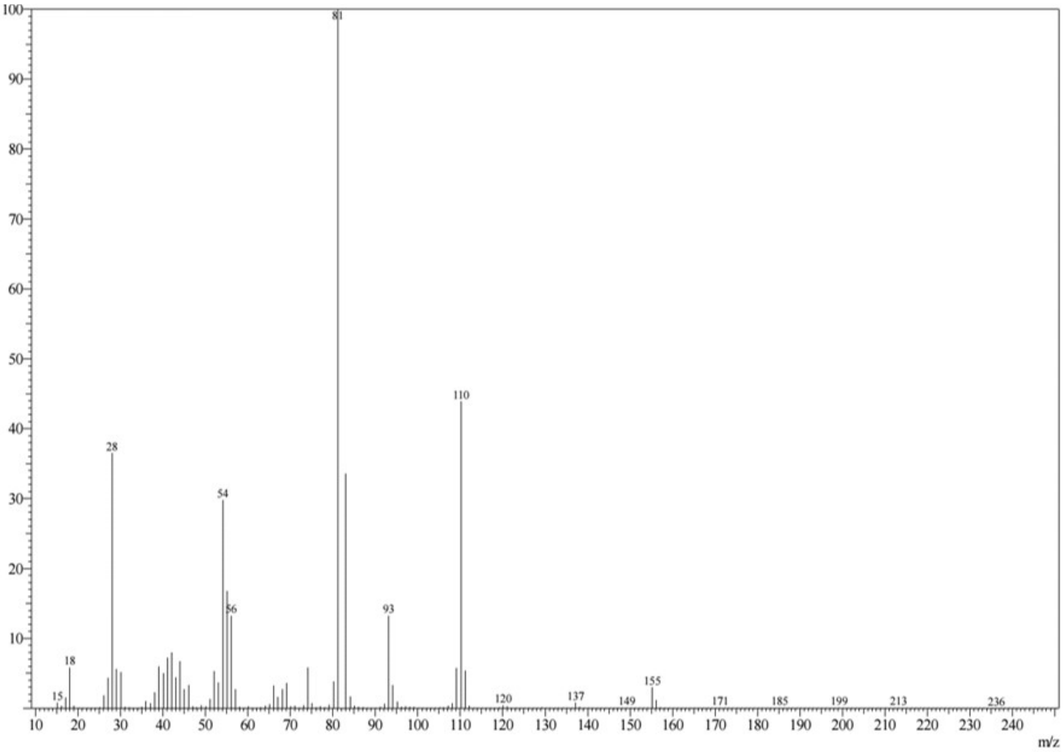

(b)

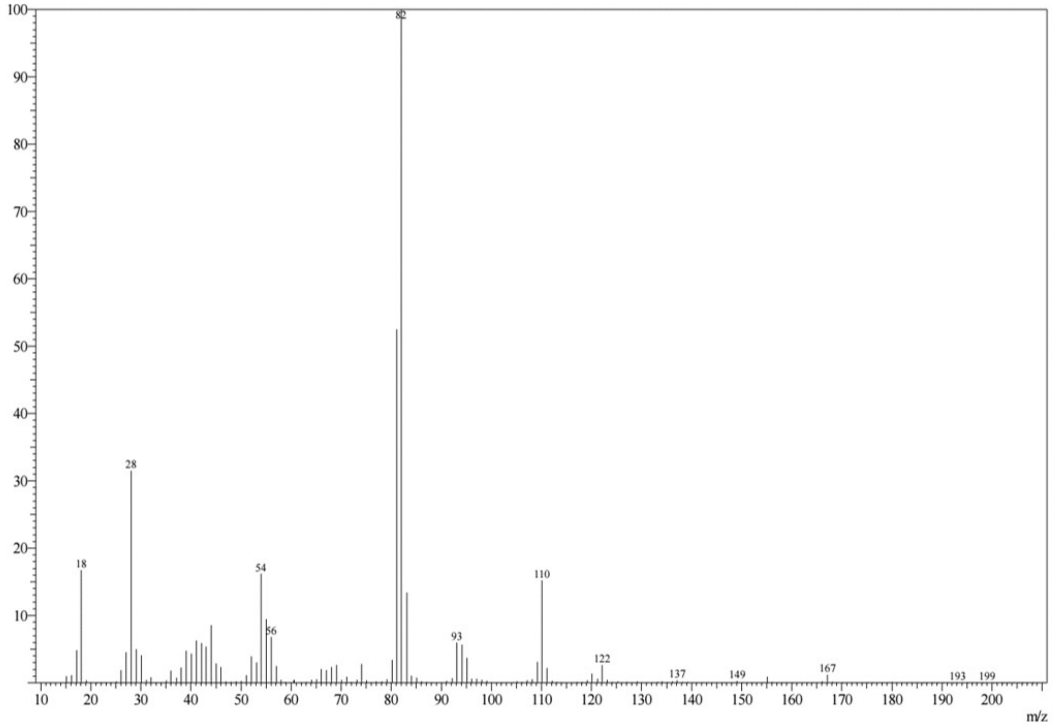

Figure S13. The EI mass spectra of His (a) and His-AuNCs (b).

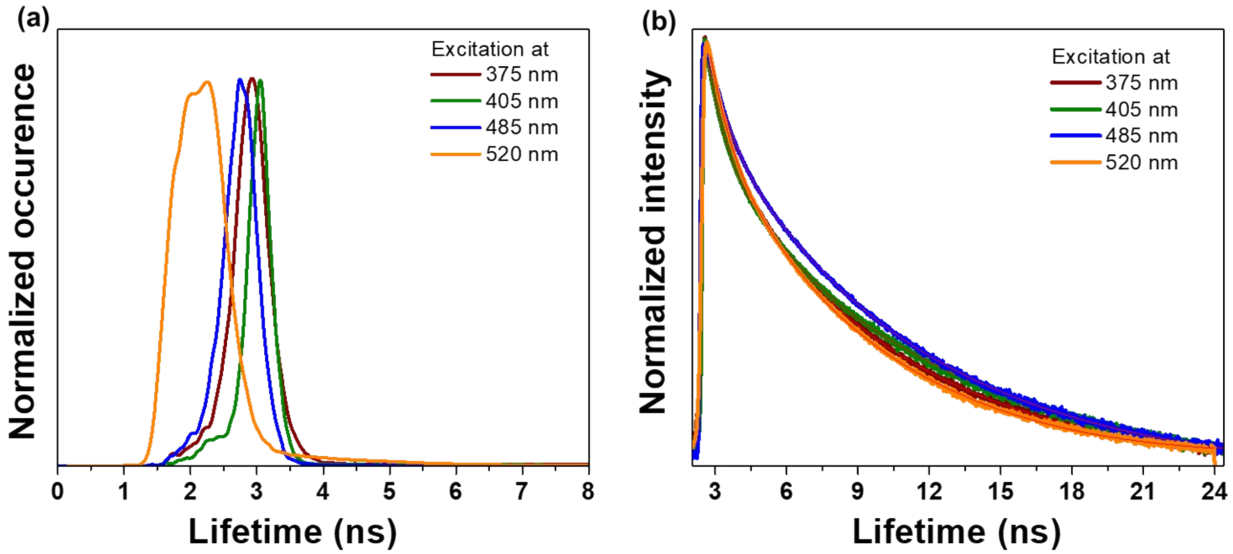

Figure S14. (a) Lifetime histograms extracted from the FLIM images recorded at different excitation wavelengths (see FLIM images in Figure 4a – bottom row). (b) Fluorescence lifetime decay curves obtained for powder His-AuNCs at different excitation wavelengths.

Table S6. The average PL lifetime obtained for His-AuNCs in solid state under different excitation wavelengths.

**Excitation (nm)**	**375**	**405**	**485**	**520**
**Average lifetime (ns)**	2.38	2.75	2.7	2.15

Average lifetime represents the intensity-weighted average lifetime calculated with the formula

$$\tau_{av\,int} = \frac{\sum_{1}^{n} a_i \tau_i^2}{\sum_{1}^{n} a_i \tau_i};$$

τ_i and a_i are the fluorescence lifetime components and corresponding amplitudes, respectively.

Table S7. The CIE color coordinates of His-AuNCs obtained at different excitation wavelengths.

Excitation (nm)	340	365	380	400	420
**CIE**	0.39/0.35	0.38/0.34	0.37/0.34	0.37/0.34	0.36/0.33

Table S8. The CIE color coordinates of His-AuNCs after thermal treatment (λ_{exc} = 365 nm).

Temperature (°C)	25	50	100	150	200
**CIE**	0.38/0.34	0.38/0.34	0.38/0.34	0.38/0.34	0.12/0.04
